# Association Between Omega‐3 Polyunsaturated Fatty Acids and Myopia: Results From the Two‐Sample and Multi‐Tissue Genomic Mendelian Randomization Study and KNHANES

**DOI:** 10.1002/fsn3.70552

**Published:** 2025-08-11

**Authors:** Yang Lu, Zhiqiang Xu, Yuzhou Wang, Shiyu Wen, Yizhou Shi, Jia Qu, Fan Lu, Liang Hu

**Affiliations:** ^1^ National Clinical Research Center for Ocular Diseases Eye Hospital, Wenzhou Medical University Wenzhou China; ^2^ State Key Laboratory of Ophthalmology, Optometry and Visual Science, Eye Hospital Wenzhou Medical University Wenzhou China

**Keywords:** docosahexaenoic acid, KNHANES, Mendelian randomization, myopia, polyunsaturated fatty acids

## Abstract

This study aims to investigate associations between omega‐3 polyunsaturated fatty acids (PUFAs) and myopia. Two‐sample Mendelian randomization (MR) was conducted to estimate the associations between plasma levels of omega‐3 PUFAs and three traits of myopia, including myopia, high myopia (HM), and refractive spherical equivalent (RSE). Summary data‐based Mendelian randomization (SMR) and colocalization analysis were conducted to examine the associations between the FADS1 and FADS2 genes and three traits of myopia in European populations. The cross‐sectional study based on the Korean National Health and Nutrition Examination Survey (KNHANES) was performed to explore the relationship in East Asian adolescents. In the Two‐sample MR study, plasma levels of total omega‐3 PUFAs (0.993[0.990, 0.996]), Docosahexaenoic acid (DHA) (0.992[0.989, 0.996]), and Eicosapentaenoic Acid (EPA) (0.969[0.955, 0.983]) were found to be significantly and inversely associated with myopia in European populations, and similar results were shown in HM and RSE. SMR (*β* = −0.028, *p* < 0.05; *p* HEIDI test > 0.05) and colocalization analysis (PPH4 = 0.926) identified an association between the expression of the FADS1 gene in the retina, crucial in PUFAs biosynthesis, and high myopia. In the cross‐sectional study, daily intake of DHA and EPA was found to be significantly associated with HM and RSE in East Asian adolescents. This study suggests a potential link between elevated omega‐3 PUFAs levels and a reduced risk of myopia, highlighting the involvement of the PUFAs biosynthesis pathway in HM among European populations. Further exploration is needed to uncover the underlying processes of this causal association.

## Graphical abstract

Our MR study indicated that omega‐3 PUFAs were found to be associated with myopia in European populations, and identified an association between the expression of FADS1 gene in retina and high myopia. In the cross‐sectional study, daily intake of DHA and EPA were found to be significantly associated with HM and RSE in East Asian adolescents. This study suggests a potential link between elevated omega‐3 PUFAs levels and a reduced risk of myopia.

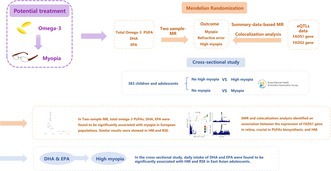

## 1 Introduction

In the past few decades, myopia, which is the most common ocular disorder, has evolved into a global societal public issue (Yam et al. 2020). The global prevalence of myopia is estimated to reach nearly 50% by the year 2050 (Holden et al. 2016), which will cause enormous economic burdens (Naidoo et al. 2019).

Current approaches for myopia control still face limitations in effectiveness and accessibility (He et al. 2015; Loh et al. 2015). Exploring a dietary supplement as a safe and accessible method to prevent or treat myopia represents a promising avenue of research. Omega‐3 polyunsaturated fatty acids (PUFAs), mainly including docosahexaenoic acid (DHA) and eicosapentaenoic acid (EPA), are vital for cardiovascular health and human neuronal development (Crawford et al. 2009). Recent studies have indicated omega‐3 PUFAs may play a role in the inhibition of myopia in animal models (Du et al. 2020; Pan et al. 2021; Mori et al. 2022). However, there is a notable absence of large‐scale, multicenter randomized controlled trials to confirm the impact of omega‐3 PUFAs supplementation on myopia.

Mendelian randomization (MR) is a statistical method using reported genetic data as instrumental variables to estimate their causal relationship between exposure and outcome (Davies et al. 2018). Compared to observational research, MR is less likely to be affected by confounding factors or reverse causality. Currently, MR has been applied in many areas of ophthalmic research, particularly in the field of myopia (Mountjoy et al. 2018; Chong et al. 2023; Li et al. 2023).

In summary, we speculate that omega‐3 PUFAs may inhibit the progression of myopia. Thus, this study seeks to explore the relationship between plasma level of omega‐3 PUFAs and three traits of myopia (myopia, high myopia, refractive spherical equivalent [RSE]) through MR, and to analyze the link between FADS genes and three traits of myopia using Summary Data‐based Mendelian Randomization (SMR) and colocalization analyses with publicly available genetic data in the European population. Further, to explore relationships among East Asian populations, it examines the relationship between omega‐3 PUFAs and myopia in adolescents aged 12–18, using data from the Korean National Health and Nutrition Examination Survey (KNHANES).

## 2 Methods

**FIGURE 1:**
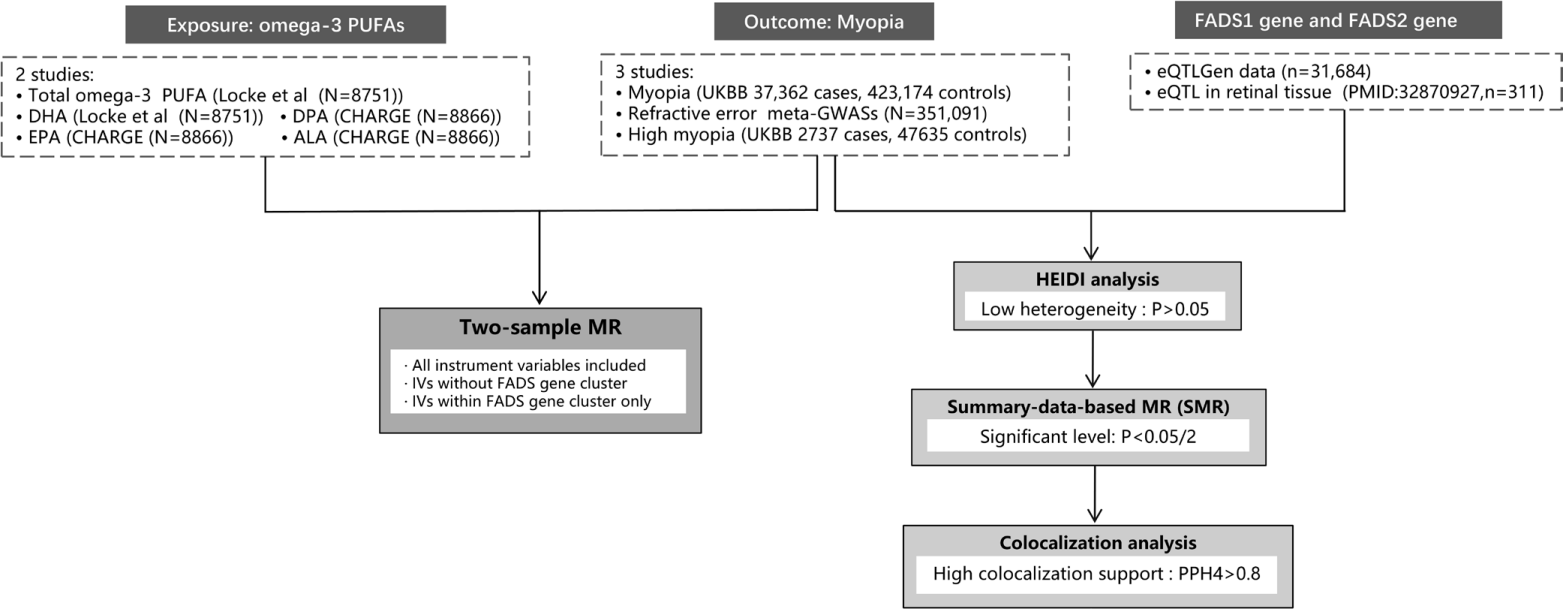
Overview of Mendelian randomization, summary data‐based Mendelian randomization (SMR), and colocalization analysis.

The study was divided into two parts: (1) to investigate the relationship between omega‐3 PUFAs and myopia traits, a two‐sample MR study was conducted. Plasma levels of PUFAs were set as exposures, and myopia, RSE, and high myopia were set as outcomes. To assess possible interactions between the FADS1 and FADS2 gene expressions and the three myopia phenotypes, SMR and colocalization analyses were used (Figure 1). (2) Due to the absence of publicly available GWAS data on myopia based on large sample sizes of East Asian populations, we conducted multivariable regression analysis using data from the KNHANES database to investigate the relationship between omega‐3 PUFAs and myopia. This study followed the specification of the Strengthening the Reporting of Observational Studies in Epidemiology Using MR and cross‐sectional studies (Tables S8 and S9).

### 2.1 GWAS Summary Statistics for Myopia Phenotype

Myopia data were downloaded from MRC Integrative Epidemiology Unit (IEU) OpenGWAS database (ukb‐b‐6353; 37,362 cases, 423,174 controls; UK Biobank) (Li et al. 2023).

RSE data were downloaded from the GWAS catalog (a meta‐analysis except for 23andMe data conducted by Hysi et al. 2020, *N* = 351,091), *β* coefficients and standard error were estimated by *z*‐score, minor allele frequency (MAF) and sample size. HM data were also downloaded from the GWAS catalog (*N* = 2737 cases, 47,635 controls; Boutin et al. 2020). The details were described in Methods S1.

### 2.2 GWAS Summary Statistics for PUFAs

To avoid the potential bias by sample overlap, we used the publicly accessible GWAS datasets for plasma levels of PUFAs from non‐UK Biobank participants of European descent, which has the great proportion of variation in that fatty acid.

GWAS Data from Metabolic Syndrome in Men (METSIM) and National FINRISK studies were used for total omega‐3 PUFAs and DHA (*N* = 8751 cases; Locke et al. 2019).

Data from the Cohorts for Heart and Aging Research in Genomic Epidemiology (CHARGE) consortium were used for DPA, EPA, and ALA (*N* = 8866 cases; Lemaitre et al. 2011).

DHA data (*N* = 8866 cases) from the CHARGE consortium was used as a replication dataset to validate the METSIM/FINRISK‐derived DHA results.

The details of the above GWAS Summary Statistics were described in Table S1 and Methods S1.

### 2.3 Selection of Fatty Acid Instrumental Variables

As a previously published MR study mentioned (Patchen et al. 2023), instrumental variables (IVs) were defined using the PLINK clumping method, with a linkage disequilibrium (LD) threshold of *r* ^2^ = 0.01 based on distinct genetic loci identified in published GWAS. The details of IVs in our study are listed in Table S2.

The FADS1 (chromosome 11: 61567099–61584475, GRCh37) and FADS2 genes (chromosome 11: 61583675–61634826, GRCh37) encode D5D and D6D enzymes, respectively, which catalyze rate‐limiting desaturase reactions in the biosynthesis of omega‐3 PUFAs (Haycock et al. 2023). To identify whether the results were driven by SNPs within the FADS region and to reduce susceptibility to horizontal pleiotropy bias (Holmes et al. 2021), a secondary analysis was conducted at the protein level by only an IV within the FADS region. Details were shown in Table S3 and Methods S1.

### 2.4 Two‐Sample MR

The MR design is based on three core assumptions (Lawlor et al. 2008). The MR analysis utilized the R statistical software along with the TwoSampleMR package (version 0.5.6). For SNPs in palindromic sequences, if the MAF is greater than 0.42, the SNP was considered uninformative and would be excluded during harmonization. Inverse variance weighted (IVW) method was used as the principal analysis, followed by MR‐Egger, weighted‐median, simple mode, weighted mode methods, and MR‐Robust Adjusted Profile Score (MR‐RAPS). Only IVW or Wald ratio was used for 2 IVs or 1 IV when MR was conducted. An association was identified as a *p* value less than 0.01 (0.05/5, Bonferroni correction). For sensitivity analysis, the Egger intercept was calculated to evaluate horizontal pleiotropy, and a heterogeneity test was also conducted.

### 2.5 Expression Quantitative Trait Locus Data

The blood data were downloaded from the eQTLGen Consortium (Võsa et al. 2021) and the retina data was downloaded from a mega analysis (Strunz et al. 2020). The SNPs within 1mb upstream and downstream of FASD1 and FADS2 gene loci were included for analysis. Details were shown in Methods S1.

### 2.6 SMR and Colocalization Analysis

We utilized the SMR tool to identify associations between the gene expression levels of FADS1 and FADS2 and the complex traits of myopia, RSE, and high myopia using summary data from eQTLs and GWAS studies. The criteria for supporting potential associations were defined as those that (1) passed the SMR *p* value with Bonferroni correction < 0.05/4, and (2) *p* value of the HEIDI test > 0.05. SMR and HEIDI methods and software tools were downloaded from Yang Lab (Zhu et al. 2016).

Colocalization analysis was conducted to evaluate the potential interactions between gene expression and traits of myopia. PPH4 > 0.8 was defined as strong evidence of colocalization while 0.8 > PPH4 > 0.5 was defined as moderate evidence of colocalization. Additionally, the probability of [PPH4/(PPH3 + PPH4)] was defined as the probability of colocalization conditional on the presence of a causal variant for outcomes (Wang et al. 2022; Bi et al. 2023). Full cis‐eQTLs of two genes were included for analysis. Details were shown in Methods S1.

### 2.7 Cross‐Sectional Study From KNHANES

We obtained data from the seventh KNHANES survey (KNHANES VII, 2016–2018) to estimate the association between omega‐3 PUFAs and three phenotypes of myopia (myopia, RSE and high myopia; Kweon et al. 2014). Participants whose characteristics lacked results of ophthalmic examination, 24‐h dietary recall, and covariates were excluded from our study. Confounding factors except for basic variables and nutritional variables were selected according to International Myopia Institute (IMI) risk factor guidelines (Morgan et al. 2021). Variables comprised refraction, omega‐3 PUFAs, and covariates including age, sex, residence, education, height, weight, body mass index (BMI), near work time (h/day), parental myopia, and weekly aerobic exercise time.

### 2.8 Statistical Analysis

Analyses were performed using R (v4.3.1) with the “survey” package to account for the complex sampling design of KNHANES. A two‐sided *p* < 0.05 defined statistical significance. Participants were stratified by myopia status (myopia/non‐myopia; high myopia/non‐high myopia). Continuous variables were compared using t‐tests or Wilcoxon rank‐sum tests, and categorical variables with chi‐square tests. Omega‐3 PUFAs (total, DHA + EPA, DHA, EPA) intakes were categorized into tertiles: T1 (< 10th percentile), T2 (10th–90th), and T3 (> 90th). Associations with myopia were assessed via multivariable logistic/linear regression, adjusted for age, sex, parental myopia, BMI, energy intake, residence, and aerobic exercise. Details about assessments and statistical analysis were shown in Methods S1.

## 3 Results

### 3.1 Two‐Sample MR Analysis

The palindromic sequence rs3798713, which served as the IV for EPA, was excluded during harmonization due to its MAF exceeding 0.42. Meanwhile, since rs187429064, which was the IV of total omega‐3 PUFAs and DHA, was not found in the GWAS of RSE, this SNP was excluded in the MR analysis when exploring the association between the level of them and RSE.

**FIGURE 2:**
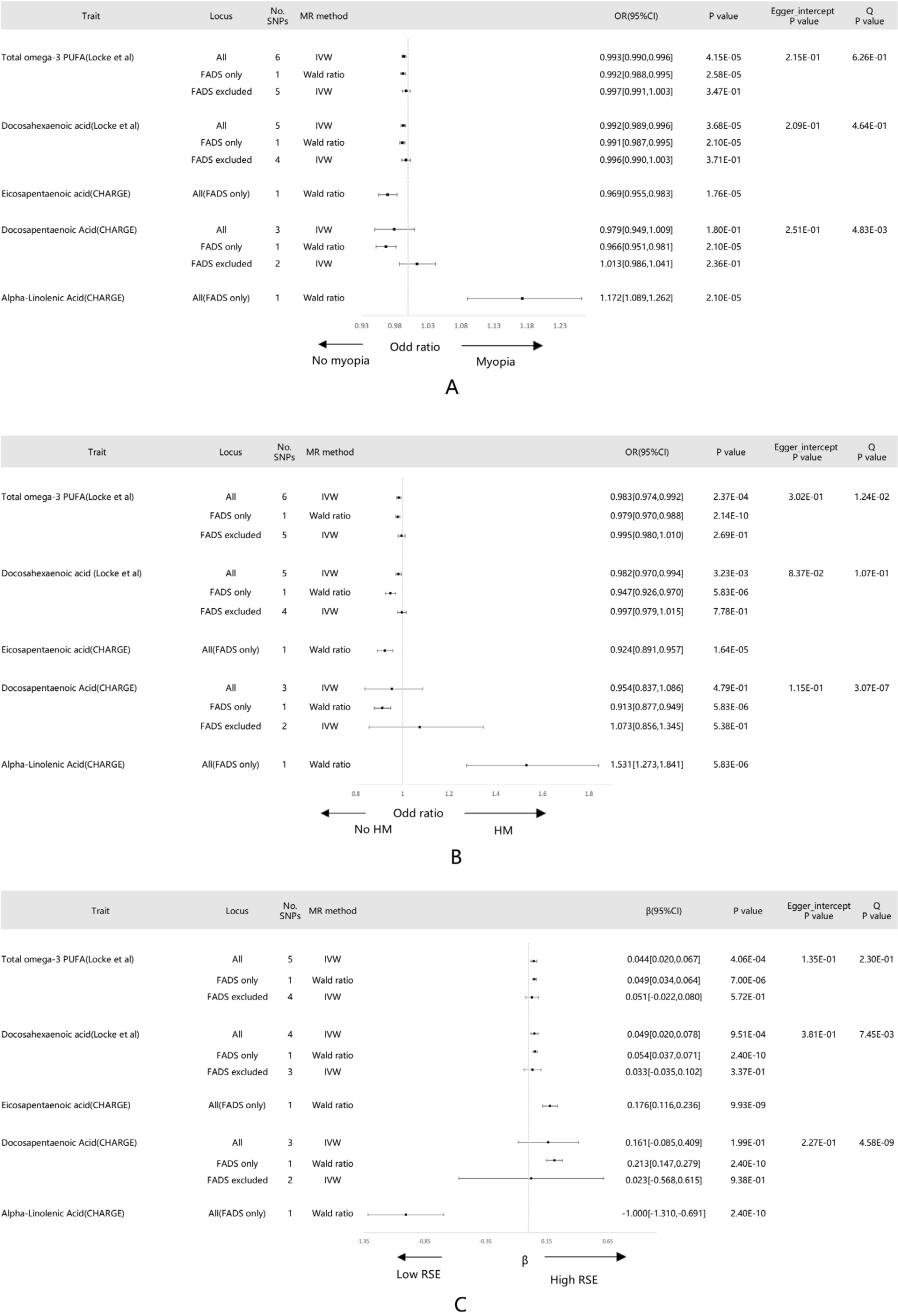
Forest plot of the results of the Mendelian randomization study investigating the effect of omega‐3 PUFAs on myopia (A), high myopia (B), refractive spherical equivalent (C).

As shown in Figure 2, although estimated effect sizes were tiny in magnitude, associations of genetically predicted total omega‐3 PUFAs and DHA with three traits of myopia were significant by IVW method (*p* < 0.01), and other methods also showed effects in the same direction as IVW (details in Table S4). Due to only one SNP regarded as IV, associations of genetically predicted EPA and ALA with three traits of myopia were significant only by Wald ratio method (*P* < 0.01). No association of genetically predicted DPA with three traits of myopia was found by IVW method (*P* > 0.01). Different from other PUFAs, a higher level of ALA was associated with an increasing risk of myopia, high myopia, and higher SE. Associations were all significant when using IVs only within the FADS region, while IVs outside the FADS region showed no causal associations. Except for the DPA where MR‐RAPS was positive but IVW negative, all other findings showed complete concordance in positivity between the two methods. In the FADS region, the results from the replication dataset for DHA were consistent with those from METSIM/FINRISK (details in Table S4).

No statistical evidence was shown for horizontal pleiotropy in the IVs for DPA, DHA, or omega‐3 PUFAs (MR‐Egger intercept test *p*‐value > 0.05 for all outcomes). The intercept test could not be used to evaluate pleiotropy in the IVs for ALA and EPA due to the number of IVs for these two fatty acids being less than three.

### 3.2 SMR and Colocalization Analysis

From eQTLGen, 5881and 5824 cis‐eQTLs about FADS1 and FADS2 in blood were obtained, respectively. And from the Institute of Human Genetics at the University of Regensburg, 4986 and 5002 cis‐eQTLs about FADS1 and FADS2 in retina were obtained, respectively. No topSNP (*p* value less than 5 × 10–8) was found in FADS2 eQTLs from retinal tissue, so SMR analysis was not performed. All results of SMR analysis showed significant associations between the expression of two genes and the three myopia traits. However, only the associations between the expression of FADS1 in both blood and retinal tissues and the trait of myopia, as well as the association between FADS1 expression in retinal tissue and the traits of RSE and high myopia, passed the HEIDI test (*p* > 0.05).

We further conducted colocalization analysis of FASD1 and FADS2 from both blood and retinal tissue and the three traits of myopia. High support of colocalization evidence was observed between FASD1 from retinal tissue and high myopia, indicating the tissue specificity of colocalization (Table 1). In addition, although PPH4 was 0.65, the probability of [PPH4/(PPH3 + PPH4)] between FASD1 from retinal tissue and myopia was 0.938, providing some evidence for colocalization. However, the remaining genes–outcome pairs had limited evidence of colocalization.

**TABLE 1:** Summary results from summary‐data‐based Mendelian randomization (SMR) and colocalization for FADS1 and FADS2 gene.

	SMR				Colocalization analysis	
	P HEIDI analysis	Beta	*p*	topSNP	PPH3	PPH4
Myopia (ukb‐6353)						
FADS1						
eQTLGen data in blood	7.60 × 10^−2^	4.00 × 10^−3^	2.79 × 10^−4^	rs968567	1.04 × 10^−1^	1.95 × 10^−1^
eQTL in retinal tissue	1.05 × 10^−1^	−1.00 × 10^−2^	1.55 × 10^−4^	rs174537	4.30 × 10^−2^	6.51 × 10^−1^
FADS2						
eQTLGen data in blood	4.10 × 10^−2^	2.00 × 10^−3^	1.32 × 10^−4^	rs61896141	8.70 × 10^−2^	3.22 × 10^−1^
eQTL in retinal tissue	—	—	—	—	—	—
RSE (Hysi et al.)						
FADS1						
eQTLGen data in blood	1.01 × 10^−7^	−1.30 × 10^−2^	1.71 × 10^−2^	rs61896141	1.00	1.51 × 10^−9^
eQTL in retinal tissue	5.00 × 10^−2^	6.40 × 10^−2^	2.57 × 10^−7^	rs174537	9.48 × 10^−1^	5.10 × 10^−2^
FADS2						
eQTLGen data in blood	4.27 × 10^−9^	−7.00 × 10^−3^	1.76 × 10^−2^	rs968567	1.00	1.46 × 10^−9^
eQTL in retinal tissue	—	—	—	—	—	—
HM (Boutin et al.)						
FADS1						
eQTLGen data in blood	3.30 × 10^−2^	1.30 × 10^−2^	3.84 × 10^−5^	rs61896141	2.14 × 10^−1^	6.44 × 10^−1^
eQTL in retinal tissue	8.70 × 10^−2^	−2.80 × 10^−2^	3.77 × 10^−5^	rs174537	4.30 × 10^−2^	9.26 × 10^−1^
FADS2						
eQTLGen data in blood	1.00 × 10^−2^	7.00 × 10^−3^	9.44 × 10^−5^	rs968567	3.45 × 10^−1^	4.27 × 10^−1^
eQTL in retinal tissue	—	—	—	—	—	—

Abbreviations: HM, high myopia; RSE, refractive spherical equivalent.

The visualization of colocalization was shown in Figure S1.

### 3.3 A Cross‐Sectional Study From KNHANES

**FIGURE 3:**
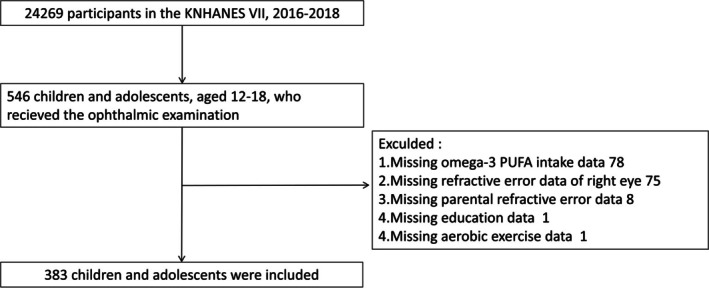
Overview of the cross‐sectional study based on Korean National Health and Nutrition Examination Survey (KNHANES).

Among 24,269 participants in KNHANES VII, 546 children and adolescents aged 12–18 years, who are most at risk of myopia development and progression at this age, received an ophthalmic examination. After participants with missing data were excluded, 383 children were included for analysis (Figure 3).

Baseline and clinical characteristics of participants divided into two groups are reported in Table S6. Among 383 participants, 326 (85.1%) were classified as high myopia and 57 (14.9%) were classified as non‐high myopia. Compared with the high myopia group and the non‐high myopia group, a significant difference was observed in BMI, weight, and SE. Details of the baseline in the myopia group and the non‐myopia group were shown in Table S5.

Due to the skewed distribution of daily intake of omega‐3 PUFAs, these variables were transformed into categorical data for analysis. Due to multicollinearity between BMI and weight, only BMI was selected in two regression models. Additionally, due to multicollinearity between DHA and EPA, they were not included together in the same model. Instead, the daily intake of DHA plus EPA was used in an independent model. Table 2 shows the results of multiple logistic regression models for the association between daily intake of omega‐3 PUFAs and high myopia. After adjustment, a significant relationship was observed between middle intake of DHA (*p* < 0.05) and risk of high myopia with reference as high intake of DHA. Table 3 showed the results of multiple linear regression models for the association between daily intake of omega‐3 PUFAs and RSE. A significant relationship was observed between middle intake of all three: DHA, EPA, DHA plus EPA (*p* < 0.05) and risk of high myopia with reference as high intake of them in multiple linear regression models. However, the association between low intake of them and high myopia was not significant in either model. No significant relationship was observed between omega‐3 PUFAs and risk of myopia (Table S7).

**TABLE 2:** Multiple logistic regression for the association between DHA, EPA, DHA, and EPA, and omega‐3 PUFAs and high myopia in Korean adolescents. Model was adjusted for age, gender, parental myopia, BMI, 24‐h intake of energy, residence, and aerobic exercise.

	Adjusted model OR (95% CI)	*p*
DHA		
T1 (< 10 percentile)	2.07 (0.44, 9.74)	0.36
T2 (≥ 10, < 90 percentile)	3.17 (1.04, 9.69)	0.04
T3 (≥ 90 percentile)	Ref	
EPA		
T1 (< 10 percentile)	0.82 (0.16, 4.14)	0.81
T2 (≥ 10, < 90 percentile)	2.17 (0.84, 5.59)	0.11
T3 (≥ 90 percentile)	Ref	
EPA and DHA		
T1 (< 10 percentile)	1.21 (0.23, 6.26)	0.82
T2 (≥ 10, < 90 percentile)	2.84 (0.90, 8.93)	0.08
T3 (≥ 90 percentile)	Ref	
Omega‐3 PUFA		
T1 (< 10 percentile)	0.77 (0.14, 4.34)	0.77
T2 (≥ 10, < 90 percentile)	1.19 (0.42, 3.41)	0.74
T3 (≥ 90 percentile)	Ref	

**TABLE 3:** Multiple linear regression for the association between intake of DHA, EPA, DHA, and EPA, omega‐3 PUFAs and RSE in Korean Adolescents. Model was adjusted for age, gender, parental myopia, BMI, 24‐h intake of energy, residence and aerobic exercise.

	Unadjusted model *β* (95% CI)	*p*	Adjusted model *β* (95% CI)	*p*
DHA				
T1 (< 10 percentile)	−1.08 (−1.94, −0.22)	0.02	−0.69 (−1.47, 0.08)	0.08
T2 (≥ 10, < 90 percentile)	−1.00 (−1.60, −0.39)	< 0.01	−0.83 (−1.38, 0.28)	< 0.01
T3 (≥ 90 percentile)	Ref		Ref	
EPA				
T1 (< 10 percentile)	−0.26 (−1.26, 0.74)	0.61	0.16 (−0.75, 1.07)	0.73
T2 (≥ 10, < 90 percentile)	−1.07 (−1.72, −0.45)	< 0.01	−0.92 (−1.48, −0.36)	< 0.01
T3 (≥ 90 percentile)	Ref.		Ref	
EPA and DHA				
T1 (< 10 percentile)	−0.76 (−1.66, 0.14)	0.10	−0.24 (−1.13, 0.66)	0.60
T2 (≥ 10, < 90 percentile)	−0.99 (−1.62, −0.38)	< 0.01	−0.83 (−1.40, −0.27)	< 0.01
T3 (≥ 90 percentile)	Ref		Ref	
Omega‐3 PUFA				
T1 (< 10 percentile)	0.26 (−0.79, 1.31)	0.63	0.58 (−0.67, 1.83)	0.37
T2 (≥ 10, < 90 percentile)	0.03 (−0.73, 0.79)	0.94	0.10 (−0.72, 0.91)	0.82
T3 (≥ 90 percentile)	Ref		Ref	

## 4 Discussion

This MR study indicated the potential inverse association between plasma levels of total omega‐3 PUFAs, DHA, EPA, and the risk of myopia based on large‐scale GWAS of three myopia traits. SMR analysis identified associations between the FADS1 gene and three myopia traits. After colocalization analysis, we discovered compelling evidence indicating that genetically predicted elevated levels of the FADS1 gene in the retina were linked to reduced risks of high myopia, showing the high tissue specificity of colocalization. In the cross‐sectional study from KNHANES, significant associations between the intake of DHA, EPA, and high myopia were further found in East Asian adolescents. Overall, the findings from the cross‐sectional study and MR study indicate the potential role of higher levels of downstream DHA and EPA in reducing the risk of high myopia, though further research is needed to confirm this relationship.

In secondary analysis for MR, we found that potential associations between omega‐3 PUFAs and three traits of myopia were almost driven by SNPs within the FADS region, especially the FADS1 region. This indicated that PUFA biosynthesis desaturase activity might play a crucial role in this relationship, which was consistent with previous studies (Jones et al. 2021; Haycock et al. 2023). A previous study also found omega‐3 PUFA effects on schizophrenia risk were mainly driven by SNPs in the FADS gene cluster. However, the results of DHA did not seem to be exclusively influenced by SNPs in the FADS gene cluster in their study (Jones et al. 2021). Additionally, in contrast to other omega‐3 PUFAs, genetically proxied PUFA desaturase activity at a high level of ALA was associated with a high risk of myopia in our study. This suggests that our results are consistent with an effect of PUFA biosynthesis on myopia, given that ALA is the upstream fatty acid, while EPA, DPA, and DHA are the downstream products of ALA.

Although SMR identified associations between the FADS1 gene and three myopia traits, strong evidence for colocalization was found only for the relationship between the FADS1 gene expressing in the retina and high myopia. Our results indicate that the FADS1 gene expressing in the retina has stronger evidence for colocalization than it does in blood. Retinal photoreceptors (cones and rods) have been reported to contain abundant DHA. NPD1, a derivative of DHA, has demonstrated anti‐inflammatory properties and roles in immune and inflammation regulation (Mukherjee et al. 2004; Marcheselli et al. 2010; Bazan et al. 2011). However, the specific role of DHA in the retina for preventing the progression of myopia remains unknown. Further studies are needed to elucidate the underlying mechanisms.

Notably, SNPs with relatively high *p* values from GWAS of RSE within the FADS1 gene region appear to segregate into two distinct groups in the visualization of colocalization (Figure S1). It seems that group one has high LD with FADS1 eQTLs from retinal tissue, while group two has extremely low LD, suggesting that there may be genes in that region other than FADS1 that influence RSE.

In the baseline description of the KNHANES cross‐sectional study, we found significant differences in BMI (*p* = 0.018) and weight (*p* = 0.008) between the high myopia group and the non‐high myopia group, consistent with findings from previous studies (Peled et al. 2022; Chen et al. 2024). In multiple linear regression analysis, although we found no significant association between levels of total omega‐3 PUFAs and high myopia, high levels of DHA and EPA were independent influencing factors of RSE and high myopia in Korean adolescents after adjustment for potential confounding factors. However, although middle intake of DHA and EPA showed significant differences when with reference to high intake in the adjusted model, no significant difference was found in low intake of them. This may be limited by the small sample size.

A cross‐sectional analysis utilizing the National Health and Nutrition Examination Survey (NHANES) database similarly demonstrated that higher dietary EPA intake was associated with reduced risk of high myopia in American adolescents aged 12–19 years, while no significant associations were observed between omega‐3 PUFA consumption and low myopia risk (Zhou et al. 2023), which are similar to the results of our study. However, myopia is widely recognized as having significant genetic determinants, particularly for high myopia. Notably, East Asian populations exhibit substantially higher prevalence rates compared to European/American populations, suggesting the research findings from Europe and America cannot be generalized to East Asia. In our study, firstly, we confirmed this association in East Asian populations (Korean cohort) with distinct genetic and environmental backgrounds, suggesting the universality of omega‐3 benefits. Secondly, our study newly identified that DHA intake independently associates with lower high myopia risk. Finally, we demonstrated a relationship between RSE reduction and higher intake of DHA and EPA; compared with the previous study, we adjusted for numerous variables according to IMI guidelines, making the results more reliable.

The difference between low myopia and high myopia can be explained by a greater contribution of genetic factors to the early development of myopia, with limited influence of diet and other environmental factors (Chua et al. 2015; Zhou et al. 2023).

There are some strengths of this study. In the MR study, several measures were conducted to ensure the robustness of the results. Three large‐scale traits of myopia were selected as outcomes for analysis, including myopia, RSE, and high myopia, and similar results were obtained among three of them. To avoid potential bias, there was no population overlap between the traits of omega‐3 PUFAs and the traits of myopia. As for the selection of IVs of omega‐3 PUFAs, they were extracted by referencing a previously published MR study, which has been proven to avoid potential horizontal pleiotropy (Patchen et al. 2023). To mitigate the impact of weak instrument variables and pleiotropy, we employed the MR‐RAPS method. Except for DPA, the results from MR‐RAPS were consistent with those of IVW. The IVs of DPA may exhibit pleiotropy, and such pleiotropy could potentially favor the development of myopia.

There are also some limitations of this study. Firstly, in our MR study, although no statistical evidence for horizontal pleiotropy was shown by the MR‐Egger intercept test and MR‐RAPS showed similar results with IVW, the expression of the FADS gene in the biosynthesis pathway may result in changes in the level of both upstream and downstream PUFAs, which led to the pleiotropy of SNP in the FADS region. For example, DPA and DHA share the same IVs in the FADS region, indicating that this SNP simultaneously affects both DPA and DHA levels. On the other hand, within the FADS region, the high LD among IVs of different PUFAs, or potential SNP sharing across these PUFAs, complicates the determination of their independent causal effects by existing statistical methods. Therefore, it is challenging to definitively determine which omega‐3 PUFAs have inhibitory effects on myopia. However, considering both the cross‐sectional study in this research and previous studies, we are more inclined to attribute this role to DHA and EPA, whose efficacy has been experimentally validated in animal models. Secondly, it is worth noting that the differences in genetic background and environment between Finnish and British/American populations (such as difference of MAF in IVs between two population, and high fish intake in Finnish population) might introduce biases in MR analyses. Therefore, we acknowledge that our MR results may not be fully generalizable to European populations. From the perspective of measurement, METSIM/FINRISK utilized nuclear magnetic resonance (NMR) spectroscopy to directly measure fatty acids without complex preparation, though it may overlook subtle structural differences. CHARGE relied on gas chromatography (GC), which precisely separates fatty acid types but requires lengthy sample processing where changes in samples may occur. To investigate the potential impact of these observed differences, we utilized DHA data from the CHARGE consortium as a replication dataset and obtained results consistent with the METSIM/FINRISK‐derived DHA associations, suggesting that potential biases may be limited. Finally, the results of the MR study do not provide information on the dosage and duration of omega‐3 PUFAs use, as mentioned in a previous study (Jones et al. 2021). In the KNHANES study, some variables, such as “light exposure” and “time outdoors” could not be included in the adjustment due to missing data in the database. Although the variables we incorporated—residence (urban/rural) and aerobic exercise time—may partially substitute for these two factors above, potential bias may still exist. Moreover, the sample size of the cross‐sectional study is relatively small, and larger studies, particularly RCTs, are needed in the future.

## 5 Conclusion

In conclusion, this MR study supported the associations between high levels of total omega‐3 PUFAs, DHA, EPA, and decreasing risk of myopia. SMR and colocalization analysis indicated the associations between the PUFAs biosynthesis pathway and high myopia in European populations. The cross‐sectional study showed the associations between DHA, EPA, and high myopia as well as RSE in East Asian adolescents. This study's findings indicate that omega‐3 PUFAs might be a potential medication for preventing myopia, especially in the application of EPA and DHA for the treatment of high myopia. However, the results may be subject to bias due to residual horizontal pleiotropy and population stratification in the MR analysis, combined with limited confounding adjustment and a relatively small sample size in the KNHANES study. Therefore, further research should be conducted to explore the application of omega‐3 PUFAs in myopia prevention.

## Author Contributions

Yang Lu: conceptualization (equal); data curation (lead); methodology (equal); writing – review and editing (equal). Zhiqiang Xu: methodology (equal); writing – review and editing (equal). Yuzhou Wang: methodology (equal); validation (equal); writing – original draft (equal). Shiyu Wen: formal analysis (equal); writing – original draft (equal). Yizhou Shi: formal analysis (equal); visualization (equal). Jia Qu: Validation (supporting); supervision (equal). Fan Lu: validation (supporting); supervision (equal). Liang Hu: funding acquisition (equal); supervision (equal).

## Conflicts of Interest

The authors declare no conflicts of interest.

## Supporting information


Data S1.

## Data Availability

The datasets supporting the conclusions of this article are available in the MRC Integrative Epidemiology Unit (IEU) OpenGWAS database (https://gwas.mrcieu.ac.uk/), GWAS catalog (https://www.ebi.ac.uk/gwas/home), cohorts for Heart and Aging Research in Genomic Epidemiology (CHARGE) consortium (https://web.chargeconsortium.com/main/results) and Institute of Human Genetics at the University of Regensburg (https://www‐huge.unix‐regensburg.de/databases.html). All codes are available upon reasonable request by contacting the corresponding author.
